# A comparison of polyethylene wear between cobalt-chrome ball heads and alumina ball heads after total hip arthroplasty: a 10-year follow-up

**DOI:** 10.1186/1749-799X-8-20

**Published:** 2013-07-08

**Authors:** ShiJun Wang, ShuDong Zhang, YuChi Zhao

**Affiliations:** 1 Department of the Joint and Bone Surgery, Yantaishan Hospital, No. 91, Jiefang Road, Yantai 264001, Shandong Province, China

## Abstract

**Study design:**

This is a retrospective study comparing polyethylene wear between ceramic ball heads and metal ball heads in total hip arthroplasty.

**Background:**

The ceramic-on-polyethylene bearing option has been introduced as an alternative to metal-on-polyethylene to minimize polyethylene wear debris and reduce subsequent osteolysis and aseptic loosening. However, the reported data were debatable. We designed this retrospective study to compare polyethylene wear between alumina ceramic ball heads and cobalt-chrome ball heads.

**Methods:**

Bilateral simultaneous primary total hip arthroplasty was performed in 22 patients between January 2002 and December 2002, with one side using metal-on-polyethylene bearing surface and the other side using alumina ceramic-on-polyethylene bearing surface. After 10 years of follow-up, the wear rate of polyethylene liner on both sides was measured using the Dorr method and compared.

**Results:**

The annual wear rate of the polyethylene liner was 0.133 mm with a standard deviation of 0.045 in the metal-on-polyethylene group and 0.056 mm with a standard deviation of 0.032 in the ceramic-on-polyethylene group. The wear rate per year was significantly lower in the ceramic-on-polyethylene group (*p* < 0.001).

**Conclusions:**

Although the implication is still controversial, our study showed that the use of ceramic head lowered the liner wear rate.

**Clinical relevance:**

Ceramic is harder and more resistant to scratching than cobalt-chrome. By increasing polyethylene liner survivorship and decreasing potential osteolytic response and aseptic loosening, ceramic head is a better alternative than cobalt-chrome head.

## Background

Polyethylene wear, periprosthetic osteolysis, and aseptic loosening are associated with the long-term success of total hip arthroplasty. The ceramic-on-polyethylene bearing option has been introduced as an alternative to metal-on-polyethylene in an attempt to minimize polyethylene wear debris and to reduce subsequent osteolysis and aseptic loosening. Ceramic is harder and more resistant to scratching than cobalt-chrome, making ceramic-on-polyethylene a theoretically more durable bearing option than metal-on-polyethylene [1-3]. Laboratory studies document dramatic reductions in wear volume, offering the prospect of increased polyethylene survivorship and less osteolytic response [4]. However, laboratory tests cannot accurately reproduce the complex environment of the human body [5]. Several *in vivo* studies reported a polyethylene liner wear rate of 0.034 to 0.071 mm/year with a 32-mm alumina head [6-9]. There were also studies which showed that ceramic heads had a lower penetration rate by 50% [10-12]. On the contrary, Sychterz et al. compared the radiological wear characteristics of 81 alumina ceramic femoral heads with a well-matched group of 43 cobalt-chrome femoral heads at a mean of 7 years. They showed that the wear of the ceramic group (0.09 mm/year) was slightly greater than that of the cobalt-chrome group (0.07 mm/year) [5].

We designed this retrospective study to evaluate the comparable performance of polyethylene liner with an alumina vs. a cobalt-chrome femoral head. The Ethical Review Committee of Yantaishan Hospital approved this study.

## Methods

Bilateral simultaneous primary total hip arthroplasty was performed in 22 patients between January 2002 and December 2002, with one side using metal-on-polyethylene bearing surface and the other side using alumina-on-polyethylene bearing surface. All operations were performed by the same surgeon (Dr. Shudong Zhang). Patients were encouraged to walk using a crutch from the third day postoperatively with partial body load. Patients started to walk with full load without a crutch 6 weeks after the operation.

The sample pool consisted of 11 women and 11 men with a mean age of 51.5 years at the operation (36–59 years). Of the sample, 2 patients were diagnosed with ankylosing spondylitis in (BASRI hip score of 4) [13], 16 patients with osteonecrosis of the femoral head (stage IV according to the Ficat classification) [14], and 4 patients with developmental dysplasia of the hip (all classified as Crowe I) [15]. All 22 patients were followed up for 10 years.

On the metal-on-polyethylene side, all patients were managed with a polyethylene liner (ENDURON, DePuy, Leeds, England), 28-mm metal head (ARTICUL/EZE, DePuy), cementless acetabular component (DURALOC, DePuy, England), and cementless femoral component (AML, DePuy). On the alumina-on-polyethylene side, the patients were managed with the same polyethylene liner, cementless acetabular component, and cementless femoral component, and with a 28-mm alumina Biolox Forte femoral head (DePuy, Warsaw, IN, USA). The polyethylene liners were 10-mm thick and were sterilized by gamma irradiation.

**Figure 1:**
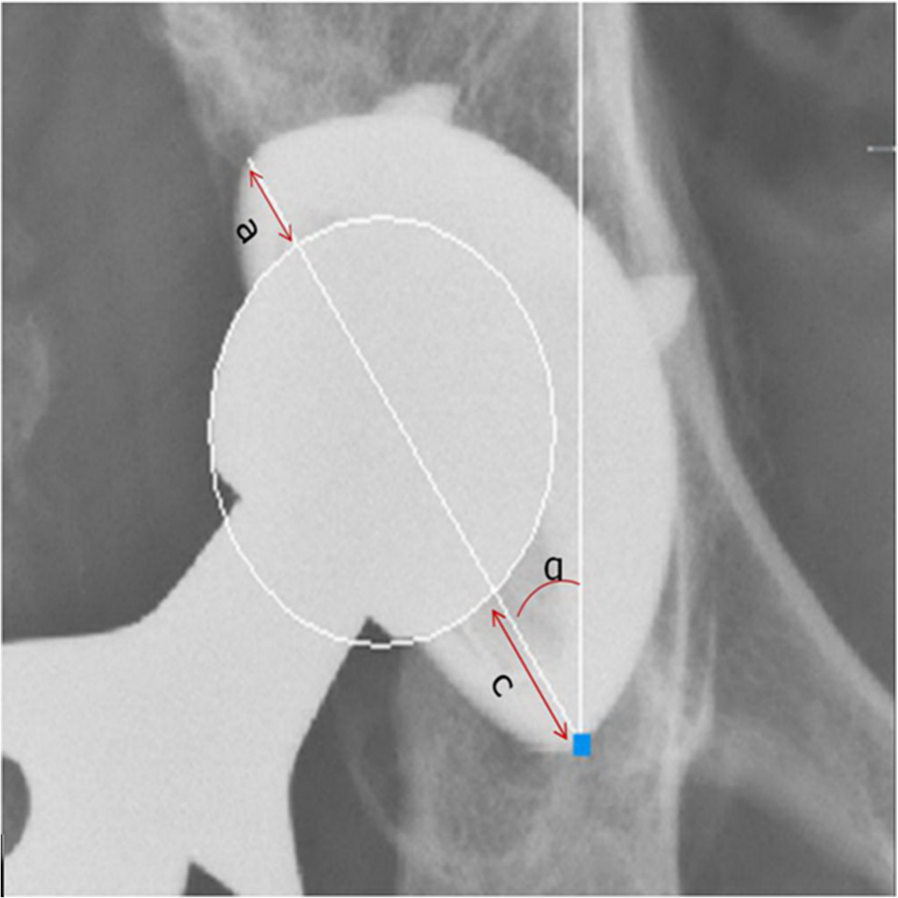
**Schematic diagram of the measurement techniques for acetabular cup wear.** A circle was drawn to determine the size of the head. A line was then drawn connecting the lateral opening of the cup, and the distances between the head and the cup (**a** and **c**) were measured.

An anteroposterior view X-ray film of the hips was taken first at the end point of follow-up using digital radiography (Radrex MRAD-A32S, Toshiba Medical Systems. Co. Ltd., Tokyo, Japan) and copied directly from the connected computer as a jpg file. Then we converted the jpg file into a dwg file which could be read by the measuring software program (Adobe Illustrator CS5, Adobe Systems Inc., San Francisco, CA, USA). Linear wear of the polyethylene liner was measured using a software program (Auto CAD 2012, AutoDesk Inc., Sausalito, CA, USA) in the dwg format. The femoral head size was determined by drawing a circle using the technique reported by Dorr and Wan [16]. A vertical line going through the center of the circle was made. A line was then drawn connecting the lateral opening of the cup, and the distances between the head and the cup were measured (Figure 1). The femoral head was measured and compared with its actual size for correction. Besides the wearing parameters, the abduction angle of the acetabular cup was also recorded. The method was described by Dorr and Wang [16]: Linear wear = (c − a) / 2.

Polyethylene wear between the metal-on-polyethylene side and alumina-on-polyethylene side was compared using a paired *t* test. The relationship between abduction angle and polyethylene wear was tested using chi-square test. All statistical analyses were performed with SPSS version 13.0 (Chicago, IL, USA). A *p* value less than 0.05 was considered significant.

## Results

No patients had postoperative dislocation of the replaced hips. No postoperative infection and breakage of the ceramic head occurred. One patient received a revise operation on the metal-on-polyethylene side due to severe wear of the polyethylene liner at the final follow-up. No hip pain and radiographic evidence of loosening of the acetabular and femoral components were observed in the other 21 patients.

The wear rate per year of the polyethylene liner in the ceramic-on-polyethylene group was 0.056 mm with 0.032 standard deviation, which was significantly less than the wear rate of 0.133 mm with 0.045 standard deviation in the metal-on-polyethylene group (*p* < 0.001). The abduction angle of the acetabular component was 46.50° with 3.949 standard deviation in the metal-on-polyethylene group and 47.05° with 5.559 standard deviation in the ceramic-on-polyethylene group. No significant difference was found (*p* = 0.680).

The abduction angle of the acetabular component in the metal-on-polyethylene group was 40°–45° in 6 patients and more than 45° in 16 patients. Polyethylene wear rate was 0.094 mm/year with 0.025 standard deviation and 0.148 mm/year with 0.041 standard deviation, respectively. The abduction angle of the acetabular component in the ceramic-on-polyethylene group was 40°–45° in 11 patients and more than 45° in 11 patients, with a polyethylene wear rate of 0.038/year with 0.003 standard deviation and 0.075 mm/year with 0.037 standard deviation (SD), respectively (Table 1).

**Table 1:** The relationship between abduction angle and liner wear rate

**Abduction angle**	**Wear rate of polyethylene liner**	***p *value**
	**(mm/year ± SD)**	
Ceramic-on-polyethylene		0.003
40°–45°	0.038 ± 0.003	
>45°	0.075 ± 0.037	
Metal-on-polyethylene		0.007
40°–45°	0.094 ± 0.025	
>45°	0.148 ± 0.041	

## Discussion

Acetabular components in total hip arthroplasty are evaluated for polyethylene wear, which is usually assessed by a radiographic technique. The techniques of Livermore, Charnley, and others were described and validated in the era of the monoblock, all-polyethylene, and cemented acetabular component, and different markers were selected [17-25]. The Dorr technique uses the opening face of the acetabular component as the reference and bases its measurements on a single radiograph [16]. Barrack et al. compared different techniques measuring metal-backed polyethylene wear with direct measurement of the retrieved polyethylene inserts in 2001 and found that the strongest correlation existed for the Dorr technique (*r* = 0.72, *p* = 0.00022) [25]. Joon Soon Kang et al. [17] modified the Dorr method and showed data comparable to that using the Devane method. This new method estimated the extent of wear to within 13.4%, with a mean error of 0.17 mm. In our study, we chose the Dorr method and combined it with computer assistance. This was a convenient and simple method to measure polyethylene wear rate. The diameter of the 28-mm ball head measured on a computer was at a mean value of about 100 using the CAD software, so the accuracy and precision of measurement were greater compared with traditional manual methods.

In our study, all patients had bilateral simultaneous total hip arthroplasty. With the same duration of follow-up, influence of primary diagnosis (?), body weight, and activity on both sides, the two sides were well matched.

Many studies have compared the wear of metal and ceramic head on polyethylene liner. Oonishi et al. reported a 0.1-mm/year rate of head penetration with alumina ceramic femoral heads compared with 0.25 mm/year with stainless femoral heads [10]. Schuller and Marti reported 0.03 mm/year of head penetration rate with alumina ceramic femoral heads compared with 0.10 mm/year with cobalt-chrome femoral heads at 9 to 11 years of follow-up [12]. Some reports showed opposite results. Sychterz et al. compared 81 alumina ceramic femoral heads with a well-matched group of 43 cobalt-chrome femoral heads at a mean of 7 years of follow-up. They showed that wear in the ceramic group was slightly greater (0.09 mm/year, SD 0.07) than that of the cobalt-chrome group (0.07 mm/year, SD 0.04). Another prospective randomized study on 70 patients with bilateral simultaneous total hip arthroplasties showed a mean annual wear of 0.17 mm with a 28-mm cobalt-chrome head compared with 0.20 mm with a 28-mm zirconia ceramic head [26]. In our study, the polyethylene liner wear rate was 0.056 mm with alumina ceramic heads, which was significantly lower than the 0.133 mm with metal heads. Our result was opposite to that in the study of Kim et al. [26], although the ceramic heads selected in their study were made from zirconia.

Besides the comparison of polyethylene wear between a metal ball head and ceramic ball head, we also studied the relationship between the abduction angle of the acetabular component and polyethylene wear. Polyethylene wear was significantly lower with abduction angle between 40° and 45° on both sides in our study. A series of studies was designed to test the hypothesis that acetabular component orientation can affect the magnitude and direction of polyethylene wear [27-29]. Using a hip wear simulator, Patil et al. [27] showed significantly different wear rates between the cups with acetabular abduction angles of 45° and 55° (mean of 17.2 compared with 21.7 mg/million cycles; *p* < 0.01). Georgiades et al. [28] studied 53 patients with congenital hip disease and found that the polyethylene wear rate was significantly greater when the cup was placed in more than 45° inclination (*p* = 0.045) or if the cup was placed lateral to the teardrop position by more than 25 mm (*p* = 0.001) after a minimum of 10 years of follow-up. Little et al. [29] found similar results in 43 uncemented hips. They showed THAs with an acetabular angle less than 45°; the mean wear rate was 0.12 mm/year (±0.01 mm/year) compared with 0.18 mm/year (±0.02 mm/year) in those with a reconstructed acetabular angle greater than 45°. Therefore, careful attention to acetabular position may minimize polyethylene wear.

In our study, only 22 patients were followed up at the end of this study. In order to get a more convictive result, more cases need to be studied.

## Conclusion

Our findings have shown that a polyethylene liner with a ceramic head yielded a significantly lower polyethylene wear rate compared with a polyethylene liner with a metal head.

## Competing interests

The authors declare that they have no competing interests.

## Authors’ contributions

SJW participated in the design of the study and performed the statistical analysis. SDZ conceived of the study, participated in the study design and coordination, and helped in drafting the manuscript. YCZ participated in the data acquisition and analysis and drafted the manuscript. All authors read and approved the final manuscript.
